# Comparison between three age-stratified cohorts reveals poor prognosis of young patients with tongue carcinoma

**DOI:** 10.3389/fonc.2022.959749

**Published:** 2022-09-02

**Authors:** Daisuke Baba, Kazuto Matsuura, Masashi Wakabayashi, Yohei Morishita, Yukio Nishiya, Wataru Okano, Toshifumi Tomioka, Takeshi Shinozaki, Ryuichi Hayashi

**Affiliations:** ^1^ Department of Head and Neck Surgery, National Cancer Center Hospital East, Kashiwa, Japan; ^2^ Department of Statistical Analysis, National Cancer Center Hospital East, Kashiwa, Japan; ^3^ Department of Surgery, The Jikei University School of Medicine, Tokyo, Japan

**Keywords:** young patients, oral tongue carcinoma, prognosis, age factors, disease-free survival

## Abstract

**Objectives:**

Investigation of the prognosis of young patients with tongue carcinoma has been the focus of several recent studies aimed at improving future precision treatment. Most studies have been two-cohort investigations comparing young and older patients, who have wide discrepancies in prognosis. Older patients, especially those aged >70 years, often have a poor general condition. This affects the prognosis of the older cohort and accounts for the discrepancies observed in two-cohort studies. Accordingly, in this study, older patients (aged ≥71 years) were separated and compared to young and middle-aged patients.

**Methods:**

A total of 257 patients with oral tongue carcinoma referred during 2011–2017 were analyzed. Patients were sorted into young (aged ≤40 years), middle-aged (aged ≥41 and ≤70 years), and older (aged ≥71 years) groups. Overall survival (OS) and disease-free survival (DFS) were compared among the groups. Furthermore, patterns of recurrence rates were compared.

**Results:**

Compared with young patients, there was no difference in OS or DFS for older patients (hazard ratio [HR]: 1.2, 95% confidence interval [CI]: 0.5–2.7 and HR: 0.7, 95% CI: 0.4–1.2, respectively) in a multivariate analysis. There was also no difference in OS (HR: 0.6, 95% CI: 0.3–1.3) for middle-aged patients. However, middle-aged patients had low recurrence rates (HR: 0.5, 95% CI: 0.3–0.8). With respect to the recurrence type, middle-aged patients had a low local recurrence rate (HR: 0.3, 95% CI: 0.1–0.7).

**Conclusion:**

Three-cohort studies should be conducted to evaluate whether the prognosis of young patients with tongue carcinoma is truly poor in terms of future precision treatment.

## Introduction

Approximately 1.5 million patients aged under 40 years are newly diagnosed with carcinoma annually, accounting for 8% of all cases (1). In 2019, lip and oral carcinomas were the 14^th^ most common of all malignant neoplasms in young individuals (2). The incidence rate of oral tongue carcinoma has been rising for the last 4 decades; this rate was higher in patients aged under 45 years than in those aged 45 years and over (3). There were approximately 30,000 cases that accounted for 2.5% of the overall incidence of malignancy and 10,000 deaths that accounted for 2.5% of deaths (2). Although the total incidence rate is not very high, the number of young patients with oral tongue carcinoma has been increasing recently in many countries, and many studies have investigated the cause of this (3–6).

The most common causes of oral tongue carcinoma are thought to be tobacco and alcohol. The total amount and frequency of tobacco or alcohol consumption are related to an increased risk of oral tongue carcinoma (7–9). However, this cumulative risk is considered low in young patients, and other causes are considered instead (4). Because of the possibly different etiologies, some investigators have thought that the prognosis of young patients with oral tongue carcinoma differs from that of older patients. HPV related oropharyngeal carcinoma has been increasing in young patients and the relation of oral tongue carcinoma with HPV has been investigated. However, HPV is not considered a main cause of this condition, unlike for oropharyngeal carcinoma including that involving the base of the tongue (10). Some authors have investigated the genetic risk factors for oral carcinoma and found some genetic changes (11–14). In the future, patients who have clinically high-risk factors for recurrence will receive an assessment to identify such genetic changes. At present, the common prognostic factors for selecting candidates for precision exams are tumor–node–metastasis (TNM) staging and extranodal extension (ENE) status. Whether age is an independent prognostic factor has been a major topic of discussion; however, there are wide discrepancies among the results of previous studies (5, 15–18). A recent systematic meta-analysis concluded that there was no difference in prognosis between patients aged ≤40 years and those aged >40 years (15). On the other hand, young patients aged <45 years present with recurrence more often than older patients aged ≥45 years, although there was no difference in the overall survival (OS) rates in another systematic review (16). However, the cause of this discrepancy has not been thoroughly discussed.

One possible reason for the discrepancy is the allocation of patients into two cohorts, young and middle-aged + older patients, without any appropriate reason, in most studies (5, 15–18). The cumulative risk of tobacco and alcohol consumption is considered higher in patients aged >40 years (4). Therefore, the application of a cut-off age around 40 years is reasonable. However, as patients get older, they are prone to more comorbidities and worse overall condition, and this restricts their treatment choices and contributes to worse prognosis, especially in patients aged >70 years (19, 20). Moreover, we believe there is no appropriate reason to combine middle-aged and older patients. We hypothesize that the discrepancy in prognosis in the two abovementioned cohort studies does not derive from the nature of the cancer itself, but from the combination of middle-aged and older patients into one cohort. The proportion of patients with more comorbidities or general decreased function among individuals belonging to older groups may affect the prognosis in middle-aged + older cohorts.

Hence, to identify the cause of the discrepancy, patients were sorted into young-adult, middle-aged, and advanced-aged groups to determine why the prognosis among them differed. The primary aim of this study was to compare overall survival (OS) and disease-free survival (DFS) in each age-stratified group. The secondary aims of this study were to investigate whether age was an independent prognostic factor, among other clinical characteristics, and compare the types of recurrence among the age groups.

## Materials and methods

### Design and patients

Clinical data of patients with oral tongue carcinoma referred to National Cancer Center Hospital East in Japan during 2011–2017 were collected retrospectively. Base of the tongue carcinoma is considered an oropharyngeal carcinoma, and to exclude the effect of HPV-related carcinoma, cases of base of the tongue carcinoma were not included in this study. The inclusion criteria were age ≥18 years, diagnosis of squamous cell carcinoma, and no prior treatment. The exclusion criteria were previous radiotherapy to the head and neck region, co-occurrence of other carcinomas, and lack of curative therapy or treatment by a therapy not recommended by the National Comprehensive Cancer Network (NCCN) guidelines (21). Co-occurrence was defined when another carcinoma was detected within 3 months before or after the time when the patient was referred to our institution.

Informed consent was obtained from all patients, and this study was approved by the Ethical Committee of the International Cancer Center (No. 2020072).

### Procedures, endpoints, and outcomes

Clinical characteristics were summarized from medical records and included age, sex, alcohol abuse, smoking status, modified Charlson Comorbidity Index, condition of patients’ teeth, clinical TNM stage, treatment choices, pathological findings, and follow-up data. Enrolled patients were sorted into the following groups: young (≤40 years); middle-aged (≥41 and ≤70 years); and older individuals (≥71 years). We adopted 40 and 70 years as cut-off values because the cut-off age for young adults has been defined as 40 years in a previous study (15), while the age of 70 years is one of the criteria used to decide whether or not chemotherapy should be administered due to issues with tolerability (19, 20). Alcohol abuse was defined as a high frequency of drinking for >5 days every week. TNM stage was defined according to the 7th edition of the staging system developed by the Union for International Cancer Control, because information about cN is somewhat lacking in the electronic record of the 8th edition.

Treatment methods were determined by a clinical oncology board consisting of head and neck surgeons, medical oncologists, and radiologists. Generally, patients with stage T1 or T2 were treated by local surgery accompanied by neck dissection when neck nodal metastasis was suspected. In advanced carcinoma cases, local resection and neck dissection were performed. In addition, reconstruction surgery was performed in the cases with advanced T stage and when verbal and swallowing disabilities were strongly predicted. Adjuvant radiotherapy ± chemotherapy was administered for pT4b or pN2c cases. In the ENE-positive cases, adjuvant therapy was not administered according to the NCCN guidelines from that time. Treatment methods could be changed in the cases where standard treatment would be intolerable due to comorbidities, decreased function, or social background based on each doctor’s decision.

To estimate the OS and disease-free survival (DFS) in each age-stratified group, OS was defined as the duration from the date of the surgery to the date of death or the last follow-up day when patients were alive or when follow-up was discontinued. DFS was defined as the duration from the date of the surgery to the date when recurrence was detected or the last follow-up day when patients were free from recurrence. OS, DFS, and the different types of recurrence were compared in the different age groups.

### Statistical analyses

Kaplan–Meier curves of OS and DFS sorted by age group were depicted. A hazard ratio (HR) with a 95% confidence interval (CI) was calculated with the Cox regression model among each clinical characteristic in the univariate analysis. Age and other variables with a significance level of <0.2 in the univariate analysis were used in a stepwise multivariate Cox regression model analysis. The cumulative incidence of the types of recurrence was also estimated. In the cumulative incidence analysis, events of interest were defined as the types of recurrence of interest, and competing events were defined as other types of recurrence and death. An HR with a 95% CI was calculated with the Fine–Gray model among each clinical characteristic in the univariate analysis. Variables with a significance level of <0.2 in the univariate analysis were used in a stepwise multivariate Fine–Gray model analysis. In this study, patients who underwent neck dissection were limited; therefore, ENE status was excluded from the multivariate analysis. cStage was also excluded from the multivariate analysis because cT and cN were relevant to cStage. P<0.05 was defined as statistically significant. Efficacy was estimated by available case analysis. All statistical analyses were performed using EZR (version 2.7-1, Saitama Medical Center, Jichi Medical University, Saitama, Japan).

## Results

This study enrolled 329 patients. We excluded 10 patients with histologies other than squamous cell carcinoma and 13 who did not receive curative therapies, including patients with distant metastases and those who did not receive recommended therapies based on NCCN guidelines. We also excluded 23 patients with recurrence, 19 with other co-occurring carcinomas, and seven who previously received radiotherapy to the head and neck area. Finally, 257 patients were included in this study.

**Figure 1:**
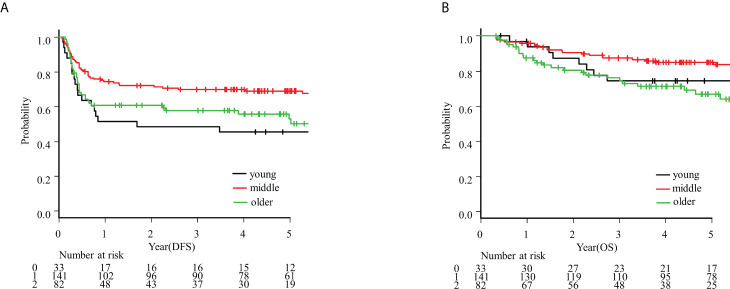
**(A)** Kaplan–Meier curve for disease-free survival (DFS); **(B)** Kaplan–Meier curve for overall survival (OS).

The characteristics of these patients are shown in **Table 1**. The median follow-up time was 4.8 years (range: 2.3–7.4). The patients’ median age was 64 years (range: 51–73). Among the age groups, the modified Charlson indexes and performance status (PS) differed significantly (p ≤ 0.001 and p<0.001, respectively); however, there were no differences in treatment methods. Alcohol abuse and tobacco consumption differed between the groups (p=0.02 and p=0.01, respectively). The status of margin findings also differed significantly (p=0.01). Kaplan–Meier curves for OS and DFS in each group are shown in **Figure 1**. The 5-year OS rates of the young, middle-aged, and older groups were 0.74, 0.85, and 0.67, respectively. The 5-year DFS rates of the young, middle-aged, and older groups were 0.46, 0.69, and 0.53 respectively.

**Table 1:** Patient characteristics.

Variables		Young	Middle-aged	Older	p	
Sex	Male	22	91	46	0.4	
	Female	11	51	36		
Alcohol abuse	–	23	67	50	0.02	
	+	8	70	30		
Tobacco	Non-smoker	15	42	41	0.01	
	Previous/current smoker	18	94	40		
Teeth	No contact to the tumor	31	114	74	0.08	
	Contact to the tumor	1	21	7		
Charlson score	0	33	119	57	<0.001	
	≥1	0	22	25		
PS	0	33	130	49	<0.001	
	≥1	0	10	32		
cT	≤2	18	71	40	0.9	
	≥3	15	71	42		
cN	0	26	93	56	0.3	
	≥1	7	49	26		
cStage	≤2	17	69	35	0.6	
	≥3	16	73	47		
ND	–	13	66	40	0.7	
	+	20	76	42		
Reconstruction	–	25	88	58	0.2	
	+	8	54	24		
Lymphatic invasion	0	29	122	66	0.3	
	≥1	3	18	16		
Vascular invasion	0	16	74	37	0.5	
	≥1	16	66	45		
Perineural invasion	0	22	104	63	0.9	
	≥1	8	36	19		
Margin status	Complete	24	129	68	0.01	
	close/incomplete	9	12	13		
ENE	–	5	14	4	0.2	
	+	2	18	13		
Adjuvant therapy	–	30	133	78	0.7	
	+	3	9	4	

PS, performance status; ND, neck dissection; ENE, extranodal extension.

The Cox regression model results are shown in **Tables 2** and **3**. In the univariate analysis, PS, cT, cStage, lymphatic invasion, vascular invasion, and perineural invasion had statistically significant impacts on OS (with HRs of 2.5, 1.9, 2.2, 2.5, 3.0, and 2.0, respectively). PS, cT, cN, lymphatic invasion, vascular invasion, perineural invasion, and the different age groups were brought stepwise, and vascular invasion had independently significant impacts on OS in the multivariate analysis (HR: 2.7, 95% CI: 1.5–4.9, respectively).

**Table 2:** Univariate and multivariate Cox regression models for DFS.

		Univariate analysis			Multivariate analysis		
Variables		HR	95% CI	*p*	HR	95% CI	*p*
Sex	Female	1.3	0.9-1.9	*0.2*			
Age	Middle-aged	0.5	0.3-0.8	*0.005*	0.5	0.3-0.8	*0.005*
	Older	0.8	0.5-1.4	*0.4*	0.7	0.4-1.2	*0.2*
Alcohol abuse	+	0.8	0.5-1.2	0.2			
Tobacco	Previous/current smoker	1.0	0.6-1.5	0.8			
Teeth	Contact to the tumor	0.9	0.5-1.6	0.6			
Charlson score	≥1	1.1	0.7-1.8	0.7			
PS	≥1	1.8	1.1-2.8	0.02			
cT	≥3	1.0	0.7-1.5	1.0			
cN	≥1	1.0	0.7-1.6	0.9			
cStage	≥3	1.1	0.8-1.7	0.6			
Lymphatic invasion	≥1	2.9	1.8-4.5	<0.001	2.2	1.3-3.5	*0.002*
Vascular invasion	≥1	2.6	1.7-4.0	<0.001	2.3	1.5-3.5	*<0.001*
Perineural invasion	≥1	1.4	0.9-2.2	0.1			
Margin status	Close/incomplete	1.6	0.9-2.6	0.08			
ENE	+	3.6	1.4-9.6	0.01			
Adjuvant therapy	+	0.9	0.4-2.2	0.9			

**Table 3:** Univariate and multivariate Cox regression models for OS.

		Univariate analysis			Multivariate analysis		
Variables		HR	95% CI	*p*	HR	95% CI	*p*
Sex	Female	0.8	0.5-1.4	0.4			
Age	Middle-aged	0.7	0.3-1.5	0.3	0.6	0.3-1.3	*0.2*
	Older	1.5	0.7-3.4	0.3	1.2	0.5-2.7	*0.7*
Alcohol abuse	+	1.3	0.7-2.1	0.4			
Tobacco	Previous/current smoker	1.0	0.6-1.7	0.9			
Teeth	Contact to the tumor	1.0	0.5-2.3	0.9			
Charlson score	≥1	1.4	0.7-2.6	0.3			
PS	≥1	2.5	1.4-4.6	0.002			
cT	≥3	1.9	1.1-3.2	0.02			
cN	≥1	1.6	0.95-2.8	0.07			
cStage	≥3	2.2	1.2-3.8	0.006			
Lymphatic invasion	≥1	2.5	1.4-4.7	0.003	1.8	0.96-3.5	0.06
Vascular invasion	≥1	3.0	1.7-5.3	<0.001	2.7	1.5-4.9	<0.001
Perineural invasion	≥1	2.0	1.2-3.4	0.01			
Margin status	Close/incomplete	1.6	0.8-3.2	0.2			
ENE	+	2.1	0.8-6.0	0.2			
Adjuvant therapy	+	1.8	0.8-4.2	0.2			

With respect to DFS, PS, lymphatic invasion, vascular invasion, ENE status, and age group (young vs. middle-aged) were significant in the univariate analysis (with HRs of 1.8, 2.9, 2.6, 3.6, and 0.5, respectively). PS, lymphatic invasion, vascular invasion, perineural invasion, margin status, and age group were brought stepwise, and lymphatic invasion, vascular invasion, and age group (young vs. middle-aged) had significant independent impacts on DFS in the multivariate analysis (HR: 2.2, 95% CI: 1.3–3.5; HR: 2.3, 95% CI: 1.5–3.5; and HR: 0.5, 95% CI: 0.3–0.8, respectively).

**Figure 2:**
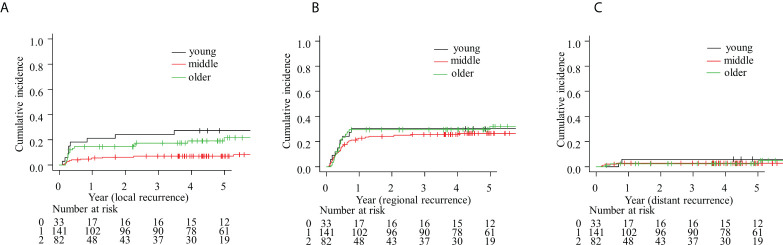
Competing risk model for **(A)** local, **(B)** regional, and **(C)** distant failure.

The age-stratified cumulative incidences of types of recurrence (local, regional, and distant) are shown in **Figure 2**. The Fine–Gray competing risk model results for local recurrence are shown in **Table 4**. In the univariate analysis, PS, margin status, perineural invasion, ENE status, adjuvant radiotherapy, and age group (young vs. middle-aged) were significant factors (HR: 2.1, 95% CI: 1.0–4.3; HR: 2.9, 95% CI: 1.5–5.8; HR: 2.3, 95% CI: 1.2–4.4; HR: 7.8, 95% CI: 1.0-60, HR: <0.001, 95% CI: 0.0–0.00007; and HR: 0.2, 95% CI: 0.1–0.5, respectively). cT, cN, PS, sex, teeth, margin status, lymphatic invasion, vascular invasion, perineural invasion, adjuvant radiation therapy, and age group were brought stepwise, and adjuvant radiation therapy, perineural invasion, and age group (young vs. middle-aged) were significant independent factors in the multivariate analysis (HR: 0.0, 95% CI: 0.0–0.00006; HR: 2.5, 95% CI: 1.3–4.8; and HR: 0.3, 95% CI: 0.1–0.7, respectively).

**Table 4:** Univariate and multivariate competing risk regression models for local recurrence.

		Univariate analysis			Multivariate analysis		
Variables		HR	95% CI	*p*	HR	95% CI	*p*
Sex	Female	1.4	0.8-2.7	0.3			
Age	Middle-aged	0.2	0.1-0.5	<0.001	0.3	0.1-0.7	0.01
	Older	0.7	0.3-1.5	0.3	0.8	0.3-1.9	0.6
Alcohol abuse	+	0.7	0.4-1.4	0.3			
Tobacco	Previous/current smoker	1.2	0.6-2.4	0.7			
Teeth	Contact to the tumor	0.4	0.1-1.6	0.2			
Charlson score	≥1	1.5	0.7-3.1	0.3			
PS	≥1	2.1	1.0-4.3	0.048			
cT	≥3	1.6	0.9-3.1	0.1			
cN	≥1	1.7	0.9-3.2	0.1			
cStage	≥3	1.8	0.9-3.5	0.07			
Lymphatic invasion	≥1	2.1	0.98-4.4	0.06			
Vascular invasion	≥1	1.6	0.9-3.1	0.1			
Perineural invasion	≥1	2.3	1.2-4.4	0.01	2.5	1.3-4.8	0.01
Margin status	Close/incomplete	2.9	1.5-5.8	0.002	1.9	0.9-4.2	0.1
ENE	+	7.8	1.0-60	0.049			
Adjuvant therapy	+	<0.001	0.00002-7.2	<0.001	<0.001	0.00002-0.00005	<0.001

## Discussion

In this study, we analyzed the OS and DFS of patients with oral tongue carcinoma sorted into three age groups. There was no difference in OS or DFS between young or older patients, including in the multivariate analysis (HR: 1.2, 95% CI: 0.5–2.7 and HR: 0.7, 95% CI: 0.4–1.2, respectively). While there was no difference in OS between the young and middle-aged groups in the univariate analysis (HR: 0.6, 95% CI: 0.3–1.3), the young patient group had fewer cases of non-recurrence than the middle-aged patient group (HR: 0.5, 95% CI: 0.3–0.8). In terms of recurrence type, there was no difference in local recurrence rates between younger and older patients in the multivariate analysis. However, there were fewer young patients without recurrence compared with middle-aged patients (HR: 0.3, 95% CI: 0.1–0.7).

Most of the published studies on the prognosis of young patients with oral carcinoma divided patients into two age cohorts and defined young patients as those aged ≤40 or 45 years (3–6, 15–18). One of the reasons for a cut-off age of 40 years was the increase in the cumulative risk of tobacco and alcohol consumption at this age (4). However, there is no absolute basis to divide patients into only two age groups when considering the differences in prognosis between young and older patients. The intensity of the treatment tends to be restricted in older patients. For example, chemoradiation may be avoided in patients aged >70 years as per the study by Pignon et al., who reported that the side effect of chemotherapy exceeds its merits for these patients in their meta-analysis (19). Besides, the modified Charlson score was higher in older patients, in whom the morbidity tended to be worse (22). As for surgery, Sanabria et al. concluded that bilateral neck dissection, reconstructive surgery, and advanced stage were prognostic factors for complications in patients aged >70 years (20), indicating that treatment strategies differed for people aged >70 years. Because the difference in treatment policy affects prognosis, it is necessary to further subdivide patients into young, middle-aged, and older groups, instead of two age groups. In this study, based on previous reports, 40 and 70 years were set as the cut-off values.

Most studies on the OS and DFS of patients with oral tongue carcinoma are two-cohort studies, comparing patients aged ≤40 or 45 years with those aged >40 or 45 years. Lee et al. reported that there was no difference in prognosis between young and older patients with oral tongue carcinoma (15). However, Jeon et al. reported that young patients have a significantly worse prognosis (17). Tagliabue et al. showed that DFS was shorter for young patients compared with that in older patients; however, OS was better in young patients (18). They also reported that young patients could tolerate more intensive treatments and had better prognoses, although they experienced more recurrence than older patients. Nevertheless, none of these studies were able to satisfactorily address the wide discrepancy in the prognoses of these age groups.

In this study, the OS and DFS of young patients were similar to those of older patients in the multivariate Cox regression analysis. Based on our hypothesis, young patients had worse prognosis than older patients due to the biological behavior of the cancer; however, other factors may make the prognosis of older patients worse. Older patients tended to receive less aggressive treatments, in terms of surgical treatment choice or adjuvant therapy, than young and middle-aged patients, due to the increased comorbidities and worse general condition. This may have resulted in a poor prognosis in older patients (23). Recently, the usefulness of evaluation tools for patient condition, such as the PS, Charlson Comorbidity Index, geriatric 8, comprehensive geriatric assessment, and Aid to Capacity Evaluation-27 index, has been reported for determining patient prognosis (24). These facts are reflected in the lack of differences in the prognoses of younger and older patients. Our results showed no differences in treatment methods between the older and other age groups despite advanced PS or modified Charlson index scores. This was because it was sometimes difficult to clearly distinguish standard and weakened therapies, because the extent to which therapies are restricted has not been standardized, and the extent of restriction was sometimes minor. In addition, older patients sometimes received standard therapy as the first treatment but then received restricted treatment when they experienced recurrence. Adjustment of these biases would be the next challenge to perform more detailed age-stratified analysis of prognosis.

The most interesting results in this study were the comparisons between young and middle-aged patients. There was no significant difference in OS between these groups; however, recurrence in young patients was more frequent than that in middle-aged patients. In these cohorts, there were no differences in treatment intensity and patient condition, and thus, this comparison would reflect the accurate biological behavior of the cancer itself and prognosis. From these results, the biological behavior of oral tongue carcinoma appears to be more aggressive in young patients, which leads to higher recurrence rates. Similar findings have been described in colorectal cancer (25). Other clinical characteristics did not affect the prognosis in either the univariate analysis or multivariate analysis. Other than clinical data, factors such as the tumor immune microenvironment or gene expression could possibly cause these differences (14, 26). A possible reason why there was no difference in OS between these groups was that the recurrent region could be controlled by systemic treatment with full intensity.

Few reports have evaluated the recurrence patterns stratified by age, although young patients are more prone to local recurrence than older patients (18). In this study, local recurrence in young patients was more frequent than that in middle-aged patients, but not in older patients. This is partly consistent with the results of previous studies. In a previous study, local recurrence of oral carcinoma was reported to be more curable than locoregional recurrence (27). In terms of oral tongue carcinoma, local recurrence is likely to be relatively curable, because the vital structures such as the skull base and carotid arteries are anatomically distant. This is supported by the fact that T4b cases in oral tongue carcinoma only accounted for 0.2% of cases, while T4b accounted for 3% of all head and neck carcinoma cases (28). This potentially causes no difference in OS between young and older patients, although the recurrence rate in the young patients was higher than that in middle-aged patients for whom the treatment options were not restricted.

These results are key for elucidation of the controversy regarding the impact of age on the prognosis of oral tongue carcinoma. Our findings proved that the degree to which treatment intensity was decreased could not be analyzed accurately, and patients’ general condition was not reflected in the treatment methods. These factors influenced the poorer prognosis of older patients. Young patients with oral tongue carcinoma would have poorer prognosis; however, the proportion of older patients with restricted treatment intensity or decreased general condition would influence the results in the two cohorts, which leads to the discrepancy of the results concerning the prognoses of young vs. older patients. It is difficult to eliminate this bias at present; hence, the age-stratified prognosis of patients with oral tongue carcinoma should be conducted in three cohorts.

There were some limitations to this study. First, this study was retrospective and had a small number of patients. Additionally, it was conducted at one institution in Japan, which reduces the generalizability of the results to other populations. In this study, the cut-off age of older patients was set as 70 years; however, age-based restriction of treatment methods varies among countries with differing average lifespans and circumstances of medical care. Thus, further age-stratified studies patients are required to determine whether a cut-off age of 70 years is appropriate worldwide. This study was conducted using TNM version 7 because the electronic medical records on clinical ENE status, apart from imaging record, are insufficient. Therefore, conversion to TNM version 8 introduced the possibility of selection bias. ENE status was only estimated based on pathological findings of neck dissection, which was not performed for all patients; therefore, ENE could not be included in the multivariate analysis. The treatments for recurrence were not analyzed in this study; therefore, treatment restriction could not be completely analyzed.

Despite these limitations, this is the first study to report age-stratified prognoses for three age groups and discuss the causes of age-related discrepancies in prognosis. Investigation of the prognosis of young patients with oral tongue carcinoma should be conducted using a separate age group for older patients aged >70 years to correctly evaluate whether the prognosis of young patients is poorer, and this should be applied in future genomic research.

## Data availability statement

The raw data supporting the conclusions of this article will be made available by the authors, without undue reservation.

## Author contributions

DB, KM, MW, YM, YN, WO, TT, TS, and RH were involved in study design and data interpretation. DB, KM, MW, and RH were involved in the data analysis. All authors critically revised the report, commented on drafts of the manuscript, and approved the final version. All authors contributed to the article and approved the submitted version.

## Acknowledgments

We appreciate Chihiro Fushimi for advising on the concepts of this article. We would also like to thank Editage for revising our manuscript.

## Conflict of interest

The authors declare that the research was conducted in the absence of any commercial or financial relationships that could be construed as a potential conflict of interest.

## Publisher’s note

All claims expressed in this article are solely those of the authors and do not necessarily represent those of their affiliated organizations, or those of the publisher, the editors and the reviewers. Any product that may be evaluated in this article, or claim that may be made by its manufacturer, is not guaranteed or endorsed by the publisher.
